# Treatment strategies for ischiofemoral impingement: a systematic review

**DOI:** 10.1007/s00167-018-5251-5

**Published:** 2018-11-13

**Authors:** Naoki Nakano, Haitham Shoman, Vikas Khanduja

**Affiliations:** Department of Trauma and Orthopaedic Surgery - Young Adult Hip Service, Addenbrooke’s-Cambridge University Hospitals NHS Foundation Trust, Hills Road, Box 37, Cambridge, CB2 0QQ UK

**Keywords:** Ischiofemoral impingement, Quadratus femoris, Endoscopy, Extra-articular impingement, Systematic review, Hip

## Abstract

**Purpose:**

There has been relatively little information about the treatment for ischiofemoral impingement (IFI) because of its rarity as well as the uncertainty of diagnosis. The aim of this study was to provide the reader with the available treatment strategies and their related outcomes for IFI based on the best available evidence, whilst highlighting classically accepted ways of treatment as well as relatively new surgical and non-surgical techniques.

**Methods:**

A systematic review of the literature from Medline, Embase, AMED, Cochrane and Google Scholar was undertaken since inception to December 2017 following the PRISMA guidelines. Clinical outcome studies, prospective/retrospective case series and case reports that described the treatment outcome for IFI were included. Animal or cadaveric studies, trial protocols, diagnostic studies without any description of treatments, technical notes without any results, and review articles were excluded.

**Results:**

This systematic review found 17 relevant papers. No comparative studies were included in the final records for qualitative assessment, which means all the studies were case series and case reports. Eight studies (47.1%) utilised non-surgical treatment including injection and prolotherapy, followed by endoscopic surgery (5 studies, 29.4%) then open surgery (4 studies, 23.5%). Mean age of the participants was 41 years (11–72 years). The mean follow-up was 8.4 months distributed from 2 weeks to 2.3 years. No complications or adverse effects were found from the systematic review.

**Conclusion:**

Several treatment strategies have been reported for IFI, and most of them have good short- to medium-term outcomes with a low rate of complications. However, there are no comparative studies to assess the superiority of one technique over another, thus further research with randomised controlled trials is required in this arena. This study explores the wide variety and categories of different treatments used for IFI to guide physicians and shed light on what can be done for this challenging cohort of patients.

**Level of evidence:**

III.

## Introduction

Ischiofemoral impingement (IFI) is an uncommon cause of pain and snapping in the hip, buttock, and groin. The pathology occurs because of a reduction of space between the lesser trochanter (LT) and the lateral border of the ischium, which leads to entrapment of the quadratus femoris (QF) muscle [37]. IFI was first described by Johnson in 1977 in three patients who had undergone an osteotomy of the hip or a hip replacement previously [18]. However, despite it being described almost 40 years ago, it is still frequently misdiagnosed or neglected because of its rarity, and the absence of specific clinical findings and diagnostic tests [14, 26]. Recently, several studies on the radiological features of IFI and distance between the ischium and the LT, i.e. ischiofemoral distance‚ have been published [14, 20, 31]. We have also recently reported on the normal ischiofemoral distance (measured as the smallest distance between the lateral cortex of ischial tuberosity and the medial cortex of the LT) and its variations using the computed tomography (CT) data of 298 normal hips and found that the mean ischiofemoral distance was 18.6 mm in females and 23 mm in males and that it increased by 1.06 mm for each 1 mm of offset and dropped by 0.09 mm with each year of age [14]. In addition, it was reported that narrowed ischiofemoral distance was associated with abnormal magnetic resonance imaging (MRI) signal intensity in the QF muscle [20, 31]. Furthermore, some studies report that the QF muscle signal changes on MRI or symptoms of IFI could be observed in patients without ischiofemoral distance narrowing (e.g. due to tumour [30] or exostosis [35, 38]), keeping the pathogenesis of IFI uncertain [20, 28].

There is relatively little information available on the best management strategy for patients with IFI. This is mainly because of the uncertainty of diagnosis and the fact that conservative treatment such as physiotherapy or activity modification is undertaken as a first step in the management of most cases with IFI [2, 26]. Surgical treatment is reserved for patients in whom conservative treatment fails. Until recently, excision of the LT with an open approach had been recommended as a normal operative technique for IFI with a narrowed ischiofemoral distance [2], however, with the improvement in arthroscopic techniques and devices, some authors report on the entire LT being accessed and resected endoscopically [10, 19, 40].

Currently, there is a lack of evidence in the literature that provides hip surgeons with evidence-based recommendations on the management of IFI, and no systematic review has been published in this arena thus far. The aim of this study, therefore, was to provide the reader with the available treatment strategies and their related outcomes for IFI based on the best available evidence, whilst highlighting classically accepted ways of treatment as well as relatively new surgical and non-surgical techniques. The objective of this systematic review would be to look at patients from both genders with no demographic restriction, who had any treatment for IFI to treat and alleviate buttock and posterior hip pain with or without distal neuropathic pain radiation and by including the studies reporting on IFI treatment and this would provide the current treatment strategies in practice.

## Materials and methods

### Search strategy

The PICOS tool was adopted to formulate the research question and modified since no comparators were sought in this study [24]. The study included randomised trials, cohort studies, case controls, and case studies as the study designs of interest. The protocol of this systematic review was developed and has been registered in the International Prospective Register of Systematic Reviews (PROSPERO 2017 CRD42017084855) [17].

Two reviewers searched the online databases (Medline, Embase, AMED, Cochrane, and Google Scholar) for the literature describing the outcomes of treatments for IFI. The Preferred Reporting Items for Systematic Reviews and Meta-Analyses (PRISMA) guidelines were used for designing this study [25]. Database search was conducted on 31st, December 2017 and retrieved articles from the databases since inception to the search date. The electronic search citation algorithm used was: [ischiofemoral (Title/Abstract) OR ischiofemoral (Title/Abstract)]. The search also included the yet to be printed search results and grey literature. Results were pooled and exported to Mendeley reference manager software (Elsevier, Amsterdam, The Netherlands) and duplicates were removed electronically and manually. The two reviewers independently reviewed all the titles and abstracts. The remaining search results were divided equally between the two reviewers and reviewed in duplicate applying the inclusion and exclusion criteria. Any discrepancies at the title and abstract stage as well as the full-text stage were resolved by consensus between the two reviewers and the third more senior author. This process led to 100% agreement between the authors.

### Study selection (inclusion and exclusion criteria)

Levels 1, 2, 3, 4, and 5 evidence (according to the Oxford Centre for Evidence-Based Medicine [29]) English language studies were eligible for inclusion in the systematic review. We excluded duplicate subject publications within separate unique studies. Both electronically published articles and print journals were included for this review. Clinical outcome studies, prospective case series, retrospective case series and case reports that described the outcomes of treatments for IFI were included. Procedures regardless of open surgery, endoscopic surgery or non-surgical treatment were included. Studies on animal models and basic science studies (e.g. cadaveric studies) were excluded. Studies describing trial protocols without any results, diagnostic studies without any description of treatments, technical notes without any results, and review articles were also excluded. The detailed inclusion and exclusion criteria are shown in Table 1.

**Table 1:** Inclusion and exclusion criteria applied to articles identified in the literature

Inclusion criteria
1. All levels of evidence
2. Written in the English language
3. Studies on humans
4. Studies reporting the outcome of treatment for ischiofemoral impingement
Exclusion criteria
1. Studies describing trial protocols without any results 2. Animal studies 3. Basic science studies (e.g. cadaveric studies) 4. Diagnostic studies without any description of treatments 5. Technical notes without any results 6. Reviews, systematic reviews

### Data extraction and analysis

Both the reviewers independently extracted the relevant study data from the final pool of included articles and recorded this data on a spreadsheet designed a priori in Microsoft Excel 2013 (Microsoft Corporation, Redmond, Washington, USA). Participant-specific demographics extracted from each study included the number of cases, gender distribution, mean age with range (years), mean length of follow-up, physical, clinical or radiological condition before the treatment, treatment strategy used in the study, final outcome, and specific comments (if any). Study-specific demographics extracted from each study included the level of evidence according to the simplified evidence level table from the Centre for Evidence-Based Medicine, Oxford, country where the study was conducted and the year of publication. Studies were then analysed and assessed using the Joanna Briggs Institute Critical Appraisal Checklist (JBICAC) for case reports and case series [17]. A scoring system was implemented based on the findings from the studies. JBICAC scores the answers to its questions as yes, no, unclear or not applicable. We then allocated numbers to each answer where domains answering with yes gets 2 points, unclear gets 1 and no gets 0. A scoring of 16 and 20 indicated the maximum points of case report and case series, respectively.

### Statistical analyses

The extracted data were then analysed using Microsoft Excel 2013. Statistical analyses in this study focused on descriptive statistics by calculating the mean values for ages and follow-up times providing an overview summary statistic of ages and follow-up times.

## Results

**Fig. 1:**
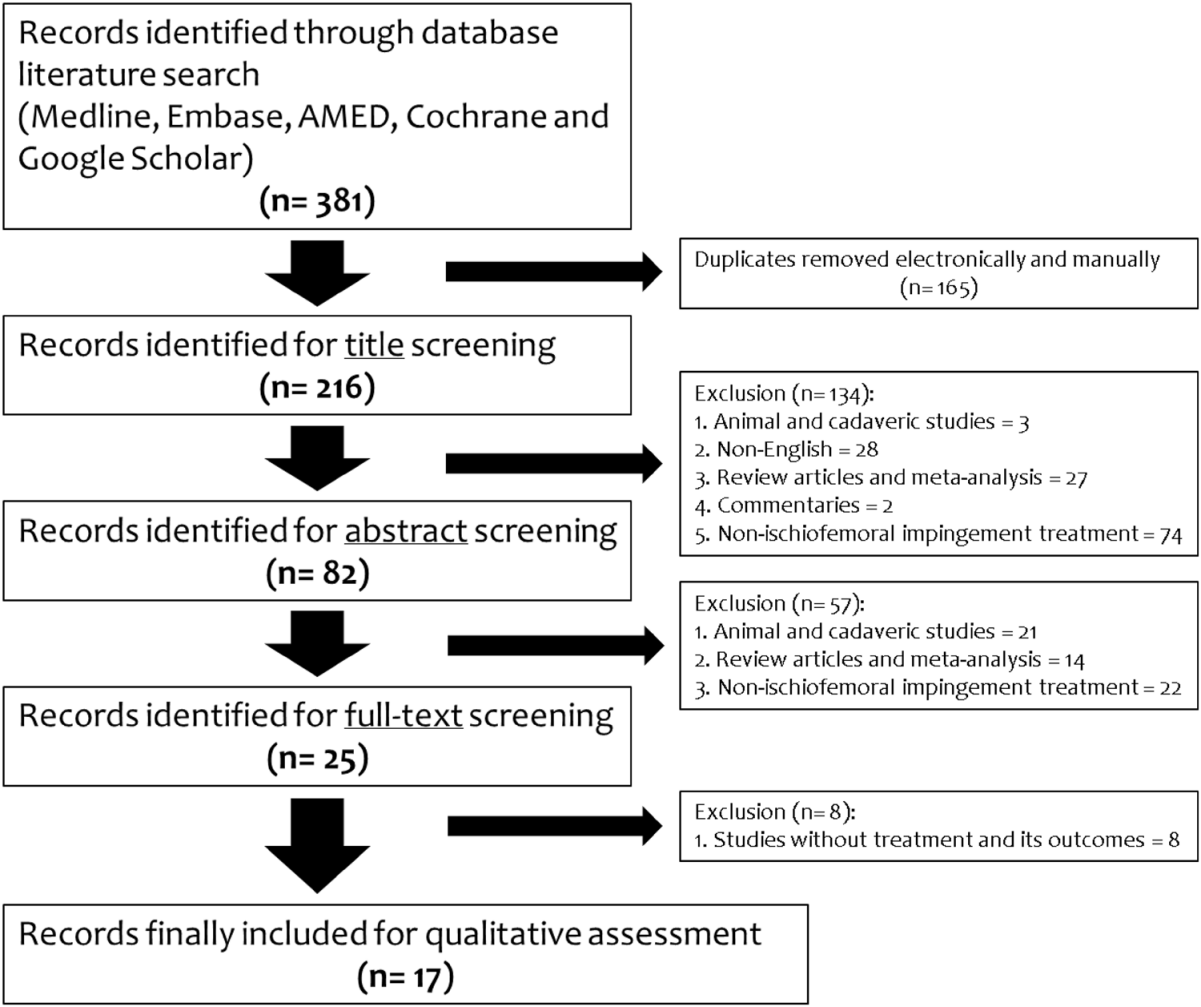
PRISMA flowchart for results of the literature database search

The initial search found a total of 381 studies from all the databases. The search process led to 100% agreement among three authors. Duplicates found were 165 articles and were removed. A total of 216 articles were then identified for title screening. One hundred and thirty-four articles were excluded based on the inclusion criteria leaving 82 articles for abstract screening. Fifty-seven articles were then excluded and 25 were included for full-text review. Eventually, 17 studies met all the inclusion criteria and were eligible for critical appraisal, quality assessment and included in the study. Of the participants, 15 (35.7%) were males and 27 (64.3%) were females (data availability: 100%). Mean age of the participants was 41 years (range 11–72 years). The mean follow-up period was 8.4 months distributed from 2 weeks to 2.3 years after the treatment. Study demographics are shown in Table 2. No complication or adverse event was found from the systematic review. The flow chart of the literature search algorithm is shown in Fig. 1. The oldest study included in this review was published in 2011. All the studies included in this systematic review were level 4 studies, which means there were no comparative studies found. Due to lack of homogeneity in studies, a meta-analysis was deemed unsuitable for this study.

**Table 2:** Demographics of the study

Parameter	
Studies analysed	17 studies
Levels of evidence: 4	17 studies (100%)
Case series	3 studies (17.6%)
Case report	14 studies (82.4%)
Participants (cases)	
Male	15 (35.7%)
Female	27 (64.3%)
Mean follow-up time (range)	8.4 months (2 weeks–2.3 years)
Mean participant age (range)	41.0 (11–72) years
Approach of treatment	
Non-surgical treatment	8 studies (47.1%)
Open surgery	4 studies (23.5%)
Endoscopic surgery	5 studies (29.4%)

Three major treatment strategies were found from this systematic review. Eight studies (47.1%) utilised non-surgical treatment including injection and prolotherapy, followed by endoscopic surgery (five studies, 29.4%) and then open surgery (four studies, 23.5%). Data extracted from all the studies are shown in Table 3. The outcomes of quality assessment using JBICAC are shown in Tables 4 and 5.

**Table 3:** Details of 17 studies included in the systematic review

References	Year	Country	LOE	Number of cases			Mean follow-up period	Age		Condition before the treatment	Treatment method	Result	Others
				Male	Female	Total		Mean	Range				
Conservative treatment													
Kim et al. [22]	2016	South Korea	4	8	6	14	2 weeks	53.4	33–72	Patients had lower buttock pain which localised at a point halfway between the lateral prominence of the greater trochanter and the ischial tuberosity corresponding to the location of the QF belly. Mean VAS before injection was 6.7 (range 3–10)	Under ultrasound guidance, 8 mL of 0.25% lidocaine was injected into the QF. The number of QF injection was: 1, five patients; 2, seven patients; 3, one patient; 4, one patient (The average frequency of injection was 2.5 times)	Two weeks after the last injection, 10 of 14 patients expressed their satisfaction as excellent or good (3 patients expressed it as fair and 1 patient expressed it as poor). There were no complications observed. At the final follow-up, mean VAS was 3.4 (range, 0–8)	
Chen et al. [3]	2017	USA	4	0	1	1	5 months	34	34	11-month history of left posterior gluteal pain. It initially presented as a dull ache in the buttock and hamstring area, exacerbated after 30 min of running or cycling, rated at NPR of 5/10 but changed to 7/10 with activity. The patient denied the history of prior trauma. Prior treatments include physical therapy, chiropractic, as well as fluoroscopically guided corticosteroid injection of the ischial bursa, sacroiliac joint, intra-articular hip joint and piriformis muscle, all without improvement	After local anaesthesia, 100 units incobotulinum toxin A reconstituted in 2.5 mL of sterile saline was injected throughout the QF	Patient’s symptoms had complete resolved and she had returned to her premorbid functional status	
Volokjina et al. [40]	2013	USA	4	0	1	1	9 months	57	57	The chronic right hip pain of 1-year duration. She described the pain as constant and deep, radiating from the lateral right groin region into the buttock, waking her up at night. Her pain was not relieved with non-steroidal anti-inflammatory drugs and was worse with athletic activities, particularly hiking. She reported no snapping symptoms	The patient received three injections into the ischiofemoral space of combinations of 3 ml of lidocaine and 40 mg Depo-medrol	After the first injection, immediate relief of symptoms lasting for 9 months was achieved. The second US-guided injection resulted in 3 weeks of symptom relief. The third injection was performed with computed tomography (CT) guidance, and the patient had continued relief for 9 months	
Kim et al. [23]	2014	South Korea	4	2	0	2	6.5 months	23.5	23–24	Initial management which consisted of NSAIDs, tramadol and physical rehabilitation for 1 month did not work. One patient had a surgical history of iliotibial band release 2 years previously and a subsequent iliopsoas tendon release 1 year previously. Preoperative VAS was 9–10/10 in both patients	The patients were treated by prolotherapy with polydeoxyribonucleotide sodium mixed with local anaesthetics injected into QF under fluoroscopic and ultrasound guidance	Patient (1) The pain intensity using the VAS decreased from 9 to 10/10 to 1–2/10, and the patient did not experience any pain for > 6 months. Follow-up MRI a month after the treatment showed that the enhancement of QF was decreased compared with that on MRI before treatment. Patient (2) The pain intensity score decreased from an initial 9–10/10 to 0–1/10, and the patient did not experience any pain for > 7 months. Follow-up MRI a month after treatment showed that the enhancement of QF was decreased compared with that on MRI before treatment	Prolotherapy refers to the injection of an irritant into a joint space, ligament, or tendon insertion site with the main aim being pain relief. Current hypotheses suggest that the presence of a local irritant may attract inflammatory mediators and possibly stimulate the release of growth factor or act as a vascular sclerosant
Hayat et al. [11]	2014	UK	4	1	0	1	1 year	16	16	The patient had an 18-month history of a dull, deep ache in his left groin, exacerbated by exercise, following an injury playing football. A plain radiograph revealed a chronic apophyseal avulsion fracture of the ischium with excessive callus formation. CT scan and MRI revealed that the bony protuberance was responsible for symptomatic IFI	Non-operative management was undertaken with painkillers as needed, rest, activity modification and physiotherapy exercise regime	Over the 12 months after treatment, the patient’s symptoms settled and he reported only a mild, infrequent ache in the groin in the final follow-up. He has resumed normal sporting activities without discomfort	
Lee et al. [24]	2013	South Korea	4	0	1	1	6 weeks	48	48	Hip MRI revealed the increased signal intensity of QF with concurrent narrowing of the ischiofemoral space. On axial T2-weighted fat-suppressed MRI, there were diffuse oedema and increased signal intensity within QF. Initial VAS was 7–8/10	NSAIDs and gabapentin were prescribed for pain relief. Hot pack, ultrasound, and interferential current therapies were applied around the hip area. The patient received an exercise program for stretching of the hip muscle and connective tissues	After 6 weeks of treatment, the pain was decreased gradually to 2–3/10 in VAS	
Yanagishita et al. [41]	2012	Brazil	4	0	1	1	3 months	31	31	Radiographic examinations demonstrated a valgus femoral neck, ischiofemoral space narrowing, and the presence of cysts in the ischium. MRI showed increased signal in QF on T2-weighted sequences	Non-surgical treatment including a taking NSAIDs for 7 days and daily physical therapy for stretching and strengthening the pelvic muscles were conducted	After 3 months of treatment, the patient showed significant functional improvement and resumed Pilates activities without any restriction	
Tosun et al. [38]	2012	Turkey	4	0	1	1	NA	11	11	The patient complained of hip and groin pain, which gradually increased during the last 2 months. MRI demonstrated narrowing of the ischiofemoral space, which was most prominent in the transverse T1-weighted sequence, and moderate oedema in QF on the fat-suppressed T2-weighted sequence	Conservative methods including rest, activity restriction, and taking NSAIDs were conducted	The patient was successfully treated conservatively	
Open surgical treatment													
Papoutsi et al. [31]	2016	UK	4	0	1	1	12 months	40	40	The patient suffered from IFI secondary to an intermuscular lipoma (2.7 × 2.6 × 0.5 cm), which was revealed on MRI and confirmed at surgery. She described the pain as a constant ache scoring 9/10 on VAS with occasional sharp shooting pains triggered by prolonged sitting and walking	The entire lipomatous tumour was excised by open surgery in the lateral position using posterior incision	The patient’s symptoms improved markedly (VAS: 0.5/10). She was able to sit without any discomfort and there was no sign of ongoing sciatic nerve irritation or IFI. The patient returned to full-time work and no longer requires any analgesia	Histology confirmed the presence of a benign intermuscularlipoma of the quadratus femoris muscle
Schatteman et al. [36]	2015	Belgium	4	1	0	1	NA	22	22	The patient suffered from groin pain, aggravating by external rotation of the hip. Standard radiographs of the hip revealed a large sessile exostosis at the medial aspect of the lesser trochanter. On MRI, a marked narrowing of the ischiofemoral space with accompanying oedema of QF was seen. Initial conservative treatment was not successful	Open resection of the exostosis was conducted	The immediate post-operative recovery was uneventful	Histological examination of the resection specimen confirmed the diagnosis of a benign cartilaginous exostosis
Viala et al. [39]	2012	France	4	0	1	1	6 months	37	37	The patient presented with hip pain of 2-year duration. Radiograph, CT, and MRI showed coxa valga and splaying of the intertrochanteric region and femoral neck as well as exostoses of the ischial tuberosity. Exostoses and femoral metaphyseal widening resulted in a narrowing of the ischiofemoral spaces	Open surgical resection of the ischial exostosis was made through an anterior approach	Six months post-operatively, hip pain was improved, appearing only after walking long distances	The patient had a past history of surgical resections of exostoses from the left knee at age 13, right knee at age 18, and right humerus at 28. At pathological examination, a typical benign exostosis was found
Ali et al. [2]	2011	UK	4	0	1	1	10 weeks	17	17	The patient showed a painful hip following an acute abduction injury to the hip while accidentally performing the splits. Seven months later, she noticed an audible and palpable clunk in her hip upon walking. MRI showed selective narrowing of the ischiofemoral space and QF space. CT-guided steroid and local anaesthetic injection around QF provided relief of her pain but not the clunking, for 24 h	Open surgical resection of the lesser trochanter was performed	The post-operative radiograph showed adequate decompression of the ischiofemoral space. At 4 weeks following the surgery, the pain had diminished to a mild discomfort and there was no clunking. At 10 weeks following surgery, the patient was asymptomatic	Before the resection of the lesser trochanter, the patient had iliotibial band Z-plasty which had no effect on the patient’s symptoms
Endoscopic surgical treatment													
Wilson et al. [41]	2016	USA	4	1	6	7	12 months	46	15–66	All patients had symptomatic, MRI-documented IFI. The preoperative scores averaged 43 points in mHHS (range 20–76 points)	The entire lesser trochanter was removed arthroscopically in the supine position	At 12 months, mHHS averaged 91 (range 76–100). There were no complications occurred. None of the patients had tenderness to palpation of the ischiofemoral space, and none had a positive IFI test or a positive long-stride walking test. None of the patients had a recurrence of their snapping, or groin or buttock pain, and all of the athletes returned to full participation in their sport	Four patients had labral tears. Two of them were repaired. Osteoplasties were performed to treat pincer impingement in two, and combined CAM and pincer deformities in four patients
Jo et al. [20]	2015	Australia	4	0	1	1	4 months	17	17	The patient complained of a 3-year history of soreness and clunking in the hip, especially in an adducted and externally rotated position. MRI revealed oedema and atrophy in the QF adjacent to the ischium, but no intra-articular pathology was observed. A plane radiograph showed a prominent anterior inferior iliac spine. The symptoms did not respond to physiotherapy, a cortisone injection and PRP treatment over a 1-year period. Psoas tendon lengthening had been performed and provided no relief of symptoms. CT-guided injection of local anaesthetic into the QF provided temporary pain relief	Endoscopic lesser trochanteric resection was conducted in the supine position	The patient’s resting pain and provocation pain on adduction and external rotation disappeared within 1 week from the operation. The symptom relief is maintained at 4-month follow-up	
Hatem et al. [10]	2015	USA	4	2	3	5	2.3 years	33.9	16–59	The mean duration of symptoms until surgery was 29.2 months (range 5–66 months). The injection was performed to rule out intra-articular pathology as a cause of posterior hip pain. The ischiofemoral and QF spaces on MRI were considered for the diagnosis. All patients had the impingement between the lesser trochanter and ischium confirmed at surgery. The mean mHHS was 51.3 (range 34.1–73.7) preoperatively. The mean preoperative VAS for pain was 6.6 (range 6–7.3)	Patients underwent endoscopic treatment with partial resection of the lesser trochanter in the supine position	The mean post-operative mHHS was 94.2 (range 78.1–100). The mean post-operative VAS for pain was 1 (range 0–4). The mean duration to return to the sport after surgery was 4.4 months (range 1–7 months). No complication was observed	Intra-articular abnormalities were observed in three patients and were treated with labral debridement, acetabuloplasty, femoroplasty, and labrum repair
Safran et al. [34]	2014	USA	4	0	1	1	2 years	19	19	The patient had oedema of QF, consistent with the diagnosis of IFI. She had undergone a hip MRI arthrogram with intra-articular anaesthetic with 95% relief of her pain. She had tried NSAIDs with some relief, and physical therapy without any benefit. The preoperative iHOT score was 32	Patients underwent endoscopic treatment with partial resection of the lesser trochanter in the supine position	At 2 years after surgery, the patient had no hip pain or involuntary snapping. On examination, she had no pain and full strength with resisted straight leg raise. Her seated hip flexion strength was 5-/5. The post-operative iHOT score was 85	
Hernandez et al. [12]	2017	Spain	4	0	2	2	6 months	43.5	42–45	Complaint of progressive, bilateral, posterior buttock pain with distal neuropathic pain radiation. On physical examination, the patient had tenderness to palpation of the ischiofemoral space and a positive long-stride walking test. Pain could be reproduced in extension, abduction and external rotation of the hip	The entire lesser trochanter was removed arthroscopically in the supine position	Patients experienced progressive improvement with immediate partial remission of their distal neuropathic radiated pain. Post-operative MRI showed a remarkable improvement of the ischiofemoral distance in both cases. Gait also improved progressively, and at the 6-month follow-up, they reported full clinical and functional recovery of the affected limb	

Age is shown in years

*LOE* level of evidence, *MRI* magnetic resonance imaging, *CT* computed tomography, *IFI* ischiofemoral impingement, *QF* quadratus femoris, *mHHS* modified Harris Hip Score, *VAS* visual analogue scale

**Table 4:** Joanna Briggs Institute Critical Appraisal Tool

Study	1. Were patient’s demographic characteristics clearly described?	2. Was the patient’s history clearly described and presented as a timeline?	3. Was the current clinical condition of the patient on presentation clearly described?	4. Were diagnostic tests or assessment methods and the results clearly described?	5. Was the intervention(s) or treatment procedure(s) clearly described?	6. Was the post-intervention clinical condition clearly described?	7. Were adverse events (harms) or unanticipated events identified and described?	8. Does the case report provide takeaway lessons?	Total score	%
*Joanna Briggs Institute critical appraisal tool for case reports* *Yes = 2/Unclear = 1/No = 0/NA*										
Ali et al. [2]	2	2	2	2	1	1	0	2	12/16	75
Chen et al. [3]	2	2	2	2	2	2	2	2	16/16	100
Hayat et al. [11]	2	2	2	2	1	1	0	2	12/16	75
Hernandez et al. [12]	2	2	2	2	2	2	2	2	16/16	100
Jo et al. [20]	2	2	2	2	2	1	0	2	13/16	81.25
Kim et al. [23]	2	2	2	2	2	2	0	2	14/16	87.5
Lee et al. [24]	2	2	2	2	1	1	0	1	11/16	68.75
Papoutsi et al. [31]	2	2	2	2	2	2	2	2	16/16	100
Safran et al. [34]	2	1	2	2	2	2	0	2	13/16	81.25
Schatteman et al. [36]	1	0	2	2	1	0	2	2	10/16	62.5
Tosun et al. [38]	2	2	2	2	1	1	0	1	11/16	68.75
Viala et al. [39]	2	2	2	2	1	1	0	2	12/16	75
Volokhina et al. [40]	2	2	2	2	2	1	0	2	13/16	81.25
Yanagishita et al. [41]	2	2	2	2	1	1	0	1	11/16	68.75

Study	1. Were there clear criteria for inclusion in the case series?	2. Was the condition measured in a standard, reliable way for all participants included in the case series?	3. Were valid methods used for identification of the condition for all participants included in the case series?	4. Did the case series have the consecutive inclusion of participants?	5. Did the case series have the complete inclusion of participants?	6. Was there clear reporting of the demographics of the participants in the study?	7. Was there clear reporting of clinical information of the participants?	8. Were the outcomes or follow-up results of cases clearly reported?	9. Was there clear reporting of the presenting site(s)/clinic(s) demographic information?	10. Was statistical analysis appropriate?	TTOTAL	%%
*Joanna Briggs Institute critical appraisal tool for case series* *Yes = 2/Unclear = 1/No = 0/NA*												
Hatem et al. [10]	2	2	2	2	2	2	2	2	2	2	20/20	100
Kim et al. [22]	2	1	1	2	2	2	2	2	2	2	18/20	90
Wilson et al. [41]	2	2	2	2	2	1	2	2	2	0	19/20	95

**Table 5:** Summary of the quality of studies within each major treatment strategy

Non-surgical treatment	
Kim et al. [22]	90
Chen et al. [3]	100
Volokjina et al. [40]	81.3
Kim et al. [23]	87.5
Hayat et al. [11]	75
Lee et al. [24]	68.8
Yanagishita et al. [41]	68.8
Tosun [38]	68.8
Mean	80
Open surgical treatment	
Papoutsi [31]	100
Schatteman [36]	62.5
Viala [39]	75
Ali [2]	75
Mean	78.1
Endoscopic surgical treatment	
Wilson [41]	95
Jo [20]	81.3
Hatem [10]	100
Safran [34]	81.3
Hernandez [12]	100
Mean	91.5

### Non-surgical treatment (eight studies)

The overall quality of the eight articles was 80% based on the JBICAC ranging from 68.75 to 100% [3, 11, 21–23, 37, 39, 41]. Four articles reported using conservative treatment (e.g. activity restriction, intake of non-steroidal anti-inflammatory drugs (NSAIDs) or physiotherapy) [11, 23, 37, 41]. Three articles reported the outcome following ultrasound (US) guided injection [21, 22, 39]. One article mentioned deploying prolotherapy with polydeoxyribonucleotide sodium mixed with the local anaesthetics under fluoroscopic and US guidance [3].

### Open surgical treatment (four studies)

The overall quality of studies was 78.1% based on the JBICAC ranging from 62.5 to 100%. The four open surgical treatment articles were subdivided based on the treatment modality [2, 30, 35, 38]. Two articles reported open surgical resection of the exostosis [35, 38]. One case report mentioned performing an open surgical resection of the LT [2]. One case report described open surgical resection of a lipomatous tumour in lateral position using a posterior incision [30].

### Endoscopic surgical treatment (five studies)

The overall quality of studies was 91.5% based on the JBICAC ranging from 81.25 to 100%. All of the five articles were endoscopic surgical resection of the LT in supine position [10, 12, 19, 33, 40]. Two articles reported on performing a partial resection of the LT [10, 33] and three articles reported on entire resection of LT [12, 19, 40].

## Discussion

The most important findings in this study are the availability of three main treatment strategies for IFI being used in current clinical practice. This study reviews all the cases of treatment for IFI (42 cases) with their results reported in the English literature and describes the outcomes of several techniques, which are divided into three categories: non-surgical treatment, open surgical treatment, and endoscopic surgical treatment. The basic pathology of IFI is that ischiofemoral space is reduced and this leads to compression of the QF muscle within the space causing pain. The QF muscle originates from the external border of the ischial tuberosity and inserts into the upper part of the linea quadrata of the proximal femur and is at risk of compression when the ischiofemoral space is reduced. The aetiology for IFI, i.e. the reason for narrowing of the ischiofemoral space, is variable, which includes ageing (muscle atrophy), female gender (increased width of pelvis), coxa profunda, coxa valga, valgus hip due to proximal femoral osteotomy, Legg–Calve–Perthes disease, total hip replacement with reduced femoral offset or medialized socket, peritrochanteric fractures with involvement of LT, abductor muscle injury causing uncompensated hip adduction during gait, and multiple or isolated exostoses [26]. The treatment in patients with IFI usually starts with conservative approaches such as rest, modification of activities and anti-inflammatory drugs such as NSAIDs. One study [33] mentioned that in their high-volume hip arthroscopy practice, only 5% of patients diagnosed with IFI required surgical intervention. The objective of this study was to discuss the outcomes of the current treatment strategies for IFI because little has been published on definitive treatment for this condition so far.

### Non-surgical treatment

Of the 17 studies found in the systematic review, five studies reported on conservative treatment [3, 11, 23, 37, 41]. The studies described the results of ‘standard’ conservative methods, e.g. the combination of rest, activity modification, taking NSAIDs and gabapentin, physiotherapy, hot packs, and ultrasound-guided injections. All the studies reported good short-term results (from 2 weeks to 1 year) without any complications. This seems to be similar to the management of other impingement syndromes wherein the first line of therapy is usually conservative, because of its less invasive approach and good patient outcomes [4]. Females also tend to have a higher incidence of IFI than males and this might be due to the anatomy of the female pelvis [18]. Females have a wider and a shallower pelvis with a more prominent LT than in males that could lead to IFI [36].

Ultrasound-guided QF muscle injection was reported to be clinically effective [21]. The anatomical location of the QF and its relation to the sciatic nerve could explain why this intervention could be useful. The sciatic nerve is closely located between the anterior surface of the gluteus maximus and the posterior surface of the QF and therefore any inflammation or spasm of this muscle will lead to irritation of the sciatic nerve. Injection of steroid, in this case, would be effective in terms of relieving the pain [34]. Another study reported that one of the ways to treat buttock pain arising from the piriformis muscle was to inject steroids and local anaesthetic [13]. Another study [21] proposed that injection of QF muscle under ultrasound guidance would be an accurate and safe procedure, as for a piriformis muscle injection, an ultrasound-guided injection was known to be more accurate than a fluoroscopically guided injection in a cadaveric model [6] and the two techniques were reported to have no difference in clinical outcomes [8]. Under ultrasound guidance, they injected 8 mL of 0.25% lidocaine into the QF muscle of 14 patients who had deep tenderness localised to a point halfway between the lateral prominence of the greater trochanter and the ischial tuberosity corresponding to the location of the QF muscle belly, and the mean pain score decreased by 49.3% in 2 weeks after the injection. They reported narrowing of the ischiofemoral space was not found in 3 of 14 patients, so their samples might include patients with other pathology, e.g. piriformis syndrome or myofascial pain syndrome.

A study [22] reported the outcome of ultrasound-guided prolotherapy with polydeoxyribonucleotide sodium for patients with IFI. Prolotherapy refers to the injection of an irritant into a specific site with the main aim being pain relief, while the mechanism is not completely understood. The presence of a local irritant might attract inflammatory mediators and possibly stimulate the release of growth factors or act as a vascular sclerosant [1, 32]. After prolotherapy, the visual analogue scale (VAS) pain score was found to be minimal (0–1/10), and follow-up MRI revealed a slightly decreased enhancement of the QF muscle compared with that before prolotherapy. They concluded that prolotherapy could be an efficacious treatment option for patients with IFI because the therapeutic effect of steroid injections has only been reported to last from 1 week to 1 month [2] while prolotherapy showed a long-term effect for > 6 months.

Injections with Botox have also been increasingly used due to its mechanism of action and improvement in patient outcomes. Botox chemodenervation acts by increasing the “space-to-content”, which may reduce muscle compression in impingement syndromes [7]. This mechanism of action was reported where Botox was used to treat chronic exertion compartment syndrome where pain faded and function improved [16].

### Open surgical treatment

Of the 17 studies found in the systematic review, four studies reported on open surgical treatment for IFI [2, 30, 35, 38]. Two of them reported on the excision of ischial exostosis [35, 38], one reported on excision of a lipomatous tumour [30], and another study described the resection of the LT [2]. The pathologic lesion was accessed by either an anterior approach or lateral approach using the trochanteric flip or through splitting of the iliotibial band.

Although no complications related to the open approach were reported, these invasive approaches can potentially endanger the neurovascular structures around the lesion, which can lead to potential delays in rehabilitation. The potential structures in danger are the medial and lateral femoral circumflex arteries, which course on the upper border of the QF muscle [27]. A cadaveric dissection study described that the medial circumflex artery was located on an average of 18 mm from the LT [9]. A very careful and meticulous approach is therefore mandatory when approaching the superior and posterior portions of the LT to avoid subsequent avascular necrosis of the femoral head. Furthermore, the resection of the LT requires detachment of the iliopsoas tendon [33], which risks persistent weakness of hip flexion.

### Endoscopic surgical treatment

Of the 17 studies in the systematic review, five studies reported on the use of endoscopic surgical management [10, 12, 19, 33, 40]. All of them reported on partial or entire resection of the LT and good short- to medium-term outcomes (from 4 months to 2.3 years) without any neurological or vascular complication. Although the endoscopic approach is useful for visualisation of the LT and ischiofemoral space, a concern that arises when using this technique is the increased risk of damaging the femoral circumflex artery as well as the perforating femoral artery which could explain why many arthroscopic surgeons have not embarked on utilising this approach [10]. Endoscopic surgery is considered as a minimally invasive surgical decompression technique with fewer complications compared with the open procedure. The psoas tendon has shown some potential for regeneration after its release following endoscopic surgery [15]. Although endoscopic surgery is performed to treat IFI caused by narrowing of the IFI space, it could also help to debride the compromised QF muscle [20].

Due to the location of the LT, the arthroscopic procedure can be approached either anteriorly or posteriorly. A study [10] described the posterior approach and reported favourable outcomes without any complications. However, at the level of the LT, the sciatic nerve is located about 4 mm from the femoral border [5], thus it can be in danger of injury unless it is approached very carefully. Another study [19] mentioned that the anterior approach was better than posterior approach because the anterior approach can avoid the need to divide the QF muscle and it minimises the risk of damage to the sciatic nerve as well as circumflex femoral vessels.

Partial resection of the LT allowed widening of the ischiofemoral space without releasing all of the iliopsoas tendon insertions, as well as a potentially decreased risk of stress fracture in comparison with complete resection [10]. This fact may be of particular important to high-performance athletes with IFI. A study [33], that reported the entire resection of the LT, partly admitted this risk by mentioning “with this patient accepting the almost assured risk of persistent hip flexor weakness”. However, another study [40] insisted that the entire LT should be removed to prevent persistence of the LT impingement due to inadequate resection of bone, which might occur with a partial resection since a thorough dynamic post-resection assessment for impingement cannot be completed with the patient in the supine position on the operating table.

The endoscopic approach seems to have many advantages when compared with the open approach especially in terms of the extent of soft tissue damage. However, care should be taken to remove as much bony debris as possible to reduce the risk of heterotopic ossification [33].

The strengths of this systematic review include the pursuit of knowledge in an important arena that has scarce published information and remains a topical subject for sports physicians and surgeons alike. The methodology is sound and encompasses a broad-based and comprehensive systematic literature search of multiple databases with multiple reviewers allowed for a very inclusive approach to capturing the vast majority of the existing literature. In addition, the included studies were critically appraised using a validated quality measurement tool [17].

Nonetheless, there are limitations which include the inclusion of English only studies and the overall low level of evidence available in the included studies on this topic (level 4 studies only). Non-prospective designs are prone to data inaccuracy as well as missing information, which subjects them to selection and source bias. Publication bias should also be recognised, and these may diminish the accuracy of the data collected and therefore limits the quality of a systematic review without a doubt.

While this current level of evidence reflects the novel and emerging nature of the treatment strategies for IFI, future studies should address comparative effectiveness of the various treatment options in this arena. Most of the studies lacked quantitative metrics in their analysis and hence quantitative conclusions could not be drawn on recommending one treatment strategy over another. To make any specific recommendations for orthopaedic surgeons with regards to treatment decisions, adequately powered long-term comparative studies focusing on two or three specific methods of treatment need to be conducted in the future.

## Conclusion

Although there are several different treatment techniques reported, the current best evidence does not support any one treatment technique as a superior method for treating IFI. There, unfortunately, remains a paucity of comparative studies, which makes it difficult to perform a meaningful assessment of the outcome of each procedure. Of the 17 studies found in the systematic review, conservative treatment as well as open/endoscopic surgical resection of the LT, were well-reported, though they were only described in limited case series and case reports.
